# Use of chest CT-scan images to differentiate between SARS-CoV-2 infection and fat embolism: A clinical case

**DOI:** 10.1016/j.radcr.2020.07.071

**Published:** 2020-07-30

**Authors:** Penance Agbelele, François Van Maris, Mario Sanguina, Bachar Zerkly, Az-Eddine Djebara, Pierre Girard

**Affiliations:** a Orthopedic Surgery and Traumatology Department, South Oise Hospital Group, Creil, France; b Medical Imaging Department, South Oise Hospital Group, Creil, France; c Orthopedic Surgery and Traumatology Department, University Hospital of Amiens-Picardy 80480, Salouel, France

**Keywords:** Thoracic CT-scan, SARS-CoV-2, Differential diagnosis, Fat embolism

## Abstract

The authors present the case of a young man victim of a traffic accident during the SARS-CoV-2 confinement, having presented a fracture of the femoral shaft that was soon complicated by respiratory failure with oxygen desaturation. In this pandemic context, Covid-19 RT-PCR tests were carried out but returned negative. The CT images could suggest either a fatty embolism, a SARS-CoV-2 infection or both. The patient's condition improved significantly after going into intensive care and only symptomatic treatment. This case demonstrates the difficulty of differential interpretation of CT images between fatty embolism and SARS-CoV-2 infection.

## Introduction

Fat embolism is a rare but serious complication of diaphyseal femoral fractures. The chest CT-scan is the imaging test of choice for making a positive diagnosis [1,2].

Since the advent of the Covid-19 epidemic, the chest CT-scan has been essential in replacing RT-PCR for the detection of pneumonia because of the moderate sensitivity of the RT-PCR test [3,4]. The CT scan characteristics of SARS-CoV-2 infection are well known and can usually be differentiated from other infectious lung diseases [3,5]. However, no study has investigated the specific features of CT scans to differentiate fat embolism syndrome from SARS-Cov-2 infection.

We report the case of a 20-year-old obese man who presented a fractured femoral shaft due to a motorcycle accident during confinement related to SARS-Cov-2. His condition was further complicated by acute respiratory failure with CT images that may suggest either SARS-Cov-2 infection or a fatty embolism or both. The difficulty lay in the possible differentiation of the 2 clinical entities when reading CT images.

## Case report

**Fig. 1:**
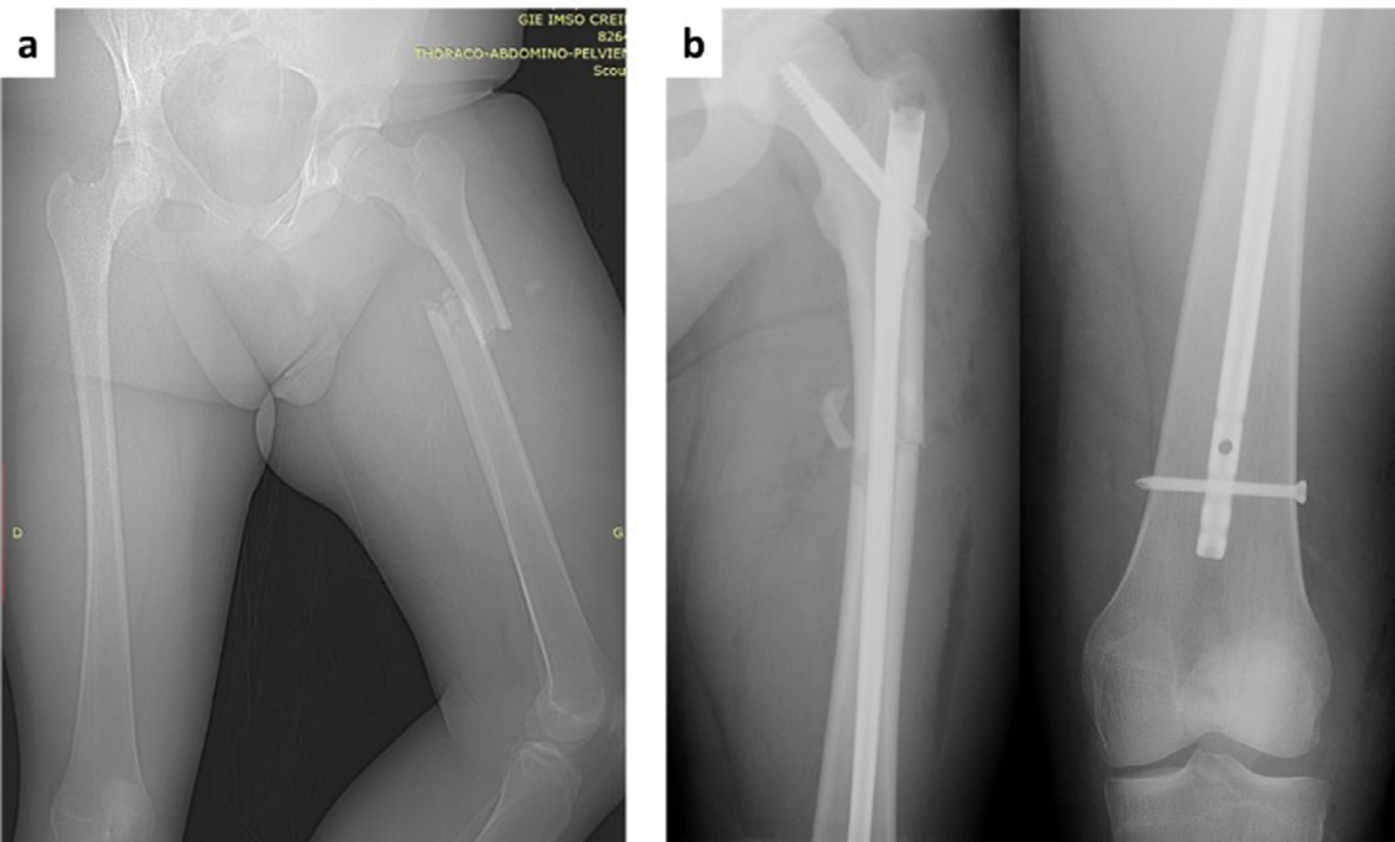
(A) Fracture of the proximal third of the left femoral shaft on computed tomography examination (scout). (B) Osteosynthesis with a long Gamma 3 nail (Stryker, Pusignan, France).

**Fig. 2:**
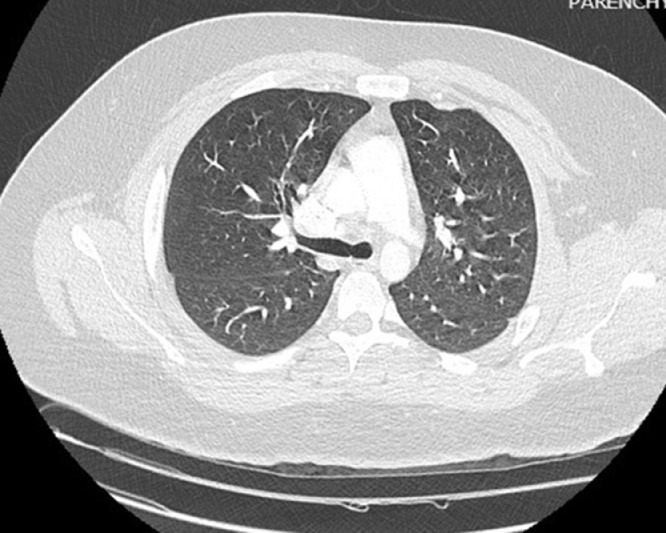
Chest CT on admission: normal lung parenchyma.

During the confinement period related to the SARS-CoV-2 pandemic, a 20-year-old obese young man was brought to the emergency room by the emergency medical services following a motorcycle accident on the public highway. He was diagnosed a fracture of the left femoral shaft which was immobilized. His Glasgow score was 15 and was hemodynamically stable. However, upon arrival, he had hyperthermia at 38° and an oxygen desaturation (SpO2) at 88%. He described a feverish state, myalgia, and nausea 4 weeks earlier, that spontaneously resolved. The radiological examinations found a fracture of the proximal third of the femoral shaft (Fig. 1A) without any associated lesions in particular of the pulmonary parenchyma (Fig. 2). The patient was considered to be potentially infected with SARS-CoV-2 due to his desaturation and hyperthermia on admission in the emergency room. Osteosynthesis using a long centromedullary nail (Fig. 1B) was performed 15 hours after admission. A first RT-PCR test performed in the operating room returned negative. The patient remained hospitalized in isolation.

**Fig. 3:**
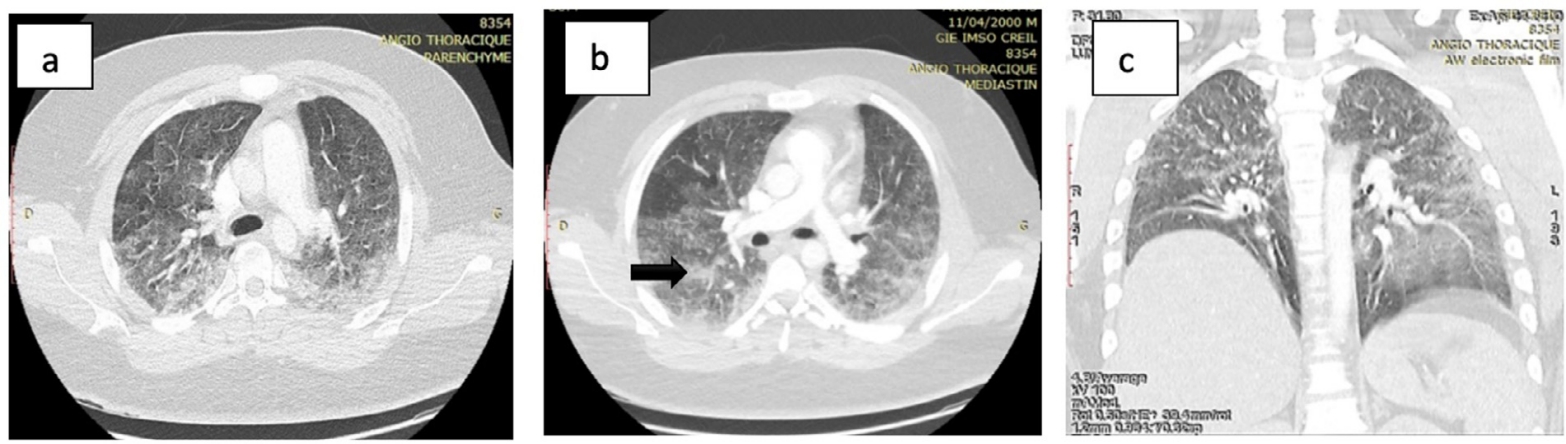
Chest CT-scan on D3. (A) Ground glass opacities and thin, bilateral reticular opacities. (B) Bilateral pulmonary condensation, posterior predominance, presence of spared healthy zones (black arrow). (C) Bilateral ground glass and thin reticular opacities affecting all the lobes.

He presented 36 hours after the intervention, still with a background of fever, an acute desaturation in oxygen (SpO2 at 70%) with polypnea (respiratory rate at 38/min) revealing an acute respiratory distress syndrome. He was hemodynamically stable. However, he had disturbances of consciousness with hallucinations. His Glasgow score was 8. He was transferred to intensive care unit. The chest CT found diffuse bilateral interstitial lung disease affecting 60% of the lung tissue and radiological signs very suggestive of a SARS-CoV-2 infection (Fig. 3). A second nasopharyngeal sample (RT-PCR) was performed which will also return negative to SARS-CoV-2. Biological findings included anemia at 8.3g/dl, CRP at 135mg/L, lymphopenia at 0.78 10^3^/mm^3^ (N:1.34-3.919), LDH at 417 (135-225), CPK at 2342U/L (39-308). Clinically, the patient had tachycardia at 122 bpm.

Following his admission on D6, while he is still hospitalized in intensive care, petechiae appeared on the chest as well as hemoptysis. Three cotton-wool spots were found in the fundus, signs in favor of fatty embolism. However, despite the negativity of RT-PCR, the diagnosis of SARS-CoV-2 infection was also retained based on clinical arguments and CT images. A third nasopharyngeal sample returned negative to Covid-19. The patient's condition has greatly improved in intensive care after a symptomatic treatment based on low molecular weight heparin and respiratory assistance. He was later transferred to a CoVid 19 department.

**Fig. 4:**
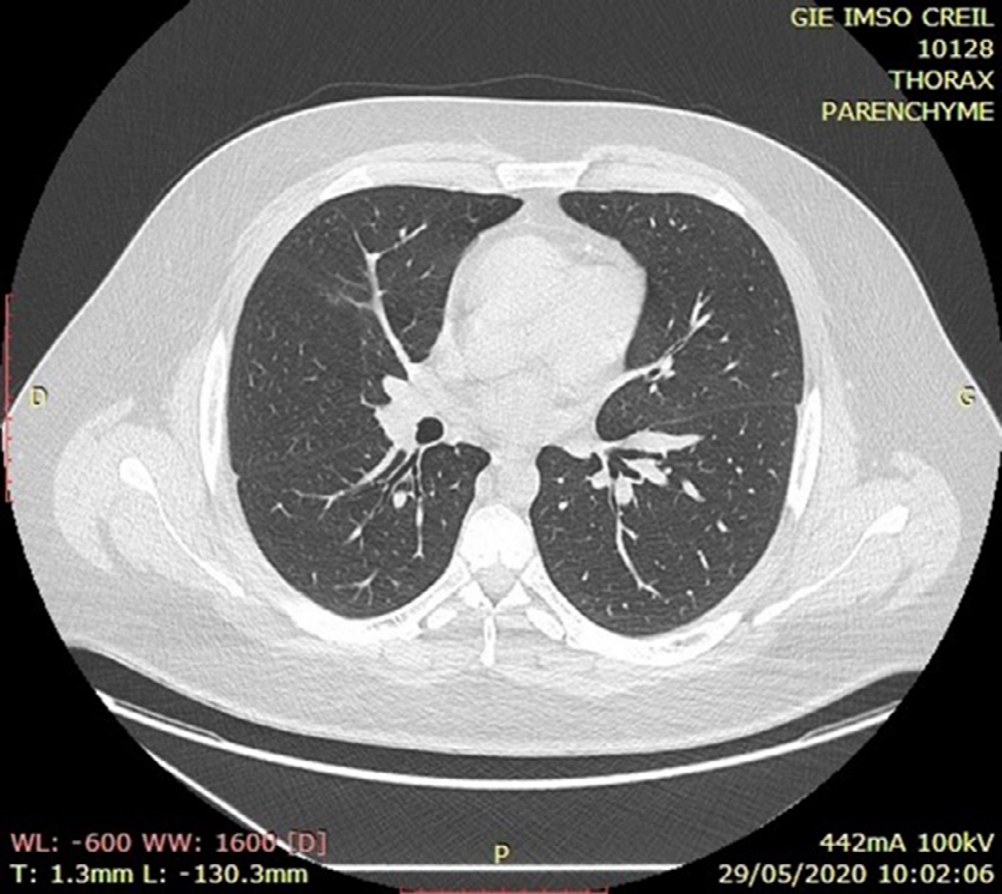
Chest CT-scan at D60: disappearance of lung lesions.

The discharge for his home took place on D21 with a good clinical and biological evolution. The thoracic CT scan performed at 2 months (Fig. 4) no longer showed any lesion of the pulmonary parenchyma. SARS-CoV-2 serology returned negative (nucleocapsid detection technique). Clinically, the patient had recovered full weight bearing painlessly and was walking with a crutch.

## Discussion

The Covid-19 pandemic started in China, then quickly spread throughout the world [6]. Medical experts set out to find reliable diagnostic means. PCR has established itself as the gold standard biological test for the diagnosis of SARS-CoV-2 infection but has a sensitivity of 56%-83% [7]. Since the main manifestation of the infection are pulmonary, the various thoracic scans carried out by several teams have made it possible to highlight recurrent characteristics, allowing radiological consensus [4].

The CT scan has become an essential diagnostic tool for Covid-19 with a sensitivity of 60%-98% and a specificity of 25 to 56% [8], [9], [10]. This moderate specificity is due to CT similarities between certain infectious, particularly pneumonia and SARS-CoV-2 pneumonia [3,5]. We could very well wonder about exploring computed tomography elements that could differentiate between coronavirus infection and FE.

Upon our patient's arrival during the pandemic, he presented with fever (38°C) and oxygen desaturation (88%) strongly suggestive of a SARS-CoV-2 infection, especially since the signs of fat embolism occur on average at H39 [11,12]. Similarly, the chest CT-scan was normal at admission, consistent with the free interval observed after the occlusion of the pulmonary arterioles in FE [1]. Similarly, with Covid-19 there is a delay of approximately 4 days between the appearance of clinical signs and those on CT images [13]. Thus, the demonstration of bilateral interstitial pneumonia at H51 does not allow discrimination.

With SARS-CoV-2 infection, pulmonary hyperdensities appear progressively, and in the last phases can lead to the appearance of “crazy paving” (intra lobar crosslinking and linear condensations) [14,15] that can also be found in some massive FEs [1].

With FE or Covid-19 pneumonia, the time at which pulmonary lesions disappear on the chest scan is still not precisely known. It can vary from 2 weeks for FE to a month for SARS-CoV-2 infection [16,17]. The development of lesions with FE can sometimes occur as calcifications associated with bronchiectasis [18], whereas with Covid-19 it occurs as late fibrosis [19]. In our case, the chest CT-scan performed at 2 months had returned to normal.

Most studies carried out on SARS-CoV-2 pneumonia highlight lesional characteristics such as ground peripheral glass opacities (sub pleural) associated with basal and posterior multilocular involvement, bilateral distribution, and enlargement of the subsegment vessels (>3 mm) [3,^4^,^10^,20]. With FE, the typical CT image also produces a ground glass image of irregular, bilateral distribution with delimitation between healthy lobular areas and injured areas, probably corresponding to variations in pulmonary perfusion due to fat emboli [2,^21^,22]. The distribution of lesions with FE is therefore more random. Through this description, we note the similarity between CT lesions found with FE and Covid-19, reflecting the complexity of differentiating between them. In addition to FE and SARS-CoV-2 infection, other less typical CT features are possible. A less common feature appearing in images of FE consists of small, poorly defined centrilobular lobules (<10 mm), with a distribution usually described as occurring in the peripheral and upper lobes and often located in the subpleural regions and along the interlobular septum [23]. Small bilateral pleural effusions are described with FE, whereas they are absent in SARS-CoV-2 infections [19].

On CT scans, in a second, post-reported reading of the image, diffuse ground glass damage with a predominance of pulmonary apices are seen, atypical in pneumonia with SARS-CoV-2 where a peripheral or circumferential distribution would be expected. This atypical distribution, however, remains compatible with SARS-CoV-2 pneumonia.

Other differential diagnoses could have been suggested when confronted with this appearance, in particular acute respiratory distress syndrome or acute pulmonary edema (however the absence of cardiomegaly and the clinical history do not favor this hypothesis).

There are also mapped spared areas of the lungs that can be encountered in SARS-CoV-2 lung diseases, but their presence in such a severe form can also be unexpected. Finally, on the middle lobe we note the presence of barely visible ill-defined micronodules, which are in themselves specific, for they are not usually visible with Covid-19 pneumonia, but can be present with FE [1,2].

In conclusion, the imaging considerations described here illustrate the problem encountered with a chest CT-scan during the Covid-19 epidemic, which is a sensitive but not very specific examination. Doubts persist regarding isolated injury from FE, infection with SARS-CoV-2, or the concurrent presence of both diseases.
